# Supplementary material for: Universal in situ oxide-based ABX3-structured seeds for templating halide perovskite growth in All-perovskite tandems
Source: Nat Commun. 2025 Feb 22;16:1894. doi: 10.1038/s41467-025-57195-w (PMC11846837; doi:10.1038/s41467-025-57195-w)
Supplement: Supplementary file 1 — Supplementary Information [file 41467_2025_57195_MOESM1_ESM.pdf]

Supplementary Information

Universal In-situ Oxide-based ABX_3-structured Seeds for Templating Halide Perovskite Growth in All-perovskite Tandems

Weiqing Chen[1,4], Shun Zhou[1,4], Hongsen Cui[1,4], Weiwei Meng[2,4], Hongling Guan[1], Guojun Zeng[1], Yansong Ge[1], Sengke Cheng[1], Zixi Yu[1], Dexin Pu[1], Lishuai Huang[1], Jin Zhou[1], Guoyi Chen[1], Guang Li[1], Hongyi Fang[1], Zhiqiu Yu[1], Hai Zhou[3], Guojia Fang[1], Weijun Ke[1]✉

[1]Key Laboratory of Artificial Micro- and Nano-structures of Ministry of Education of China, School of Physics and Technology, Wuhan University, Wuhan, China.

[2]South China Academy of Advanced Optoelectronics, South China Normal University, Guangzhou, China.

[3]International School of Microelectronics, Dongguan University of Technology, Dongguan, Guangdong, China.

[4]These authors contributed equally: Weiqing Chen, Shun Zhou, Hongsen Cui, Weiwei Meng.

✉E-mail: weijun.ke@whu.edu.cn

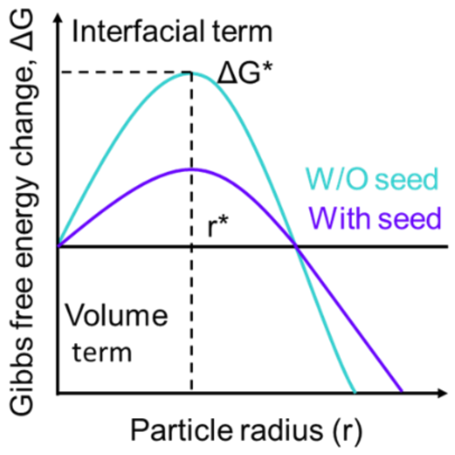

Supplementary Figure 1. Gibbs free energy diagram for perovskite nucleation with or without $PbSnO_3$ seeds[1,2].

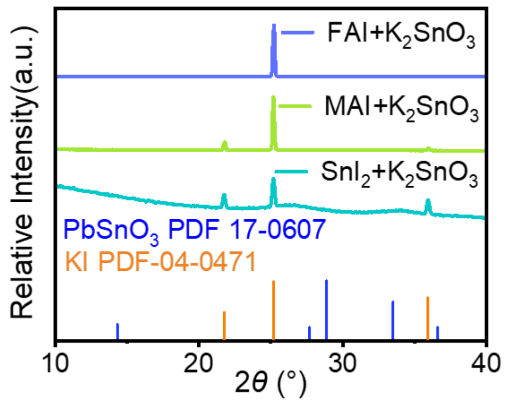

Supplementary Figure 2. XRD patterns of the products after the reaction of K_2SnO_3 with FAI, MAI, and SnI_2.

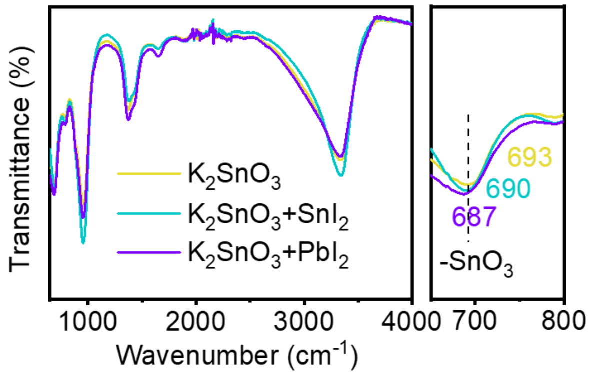

Supplementary Figure 3. FTIR spectra of K_2SnO_3, K_2SnO_3/PbI_2, and K_2SnO_3/SnI_2 complexes.

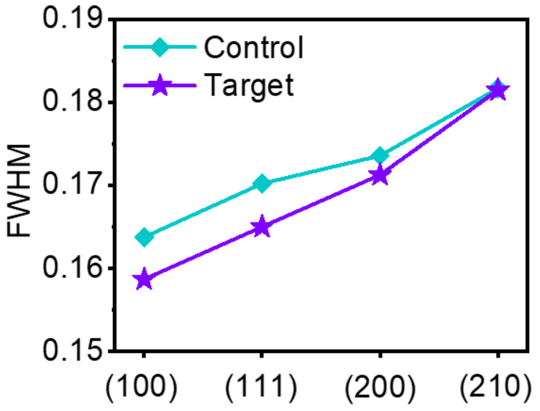

Supplementary Figure 4. FWHM of the corresponding diffraction peaks of perovskite thin films from Figure 2a.

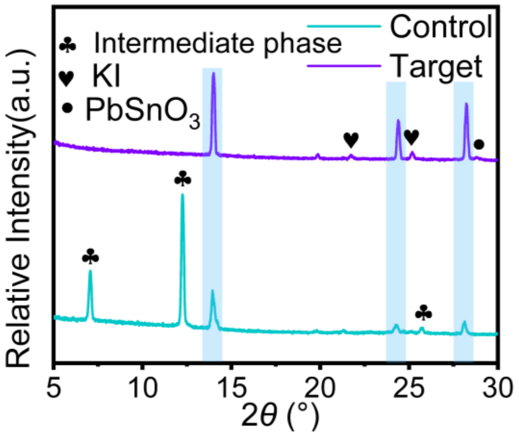

Supplementary Figure 5. XRD patterns of unannealed intermediate-phase wet films prepared with or without K_2SnO_3 treatment (The club symbol represents intermediate phase, the heart shape represents KI, and the circle represents $PbSnO_3$.).

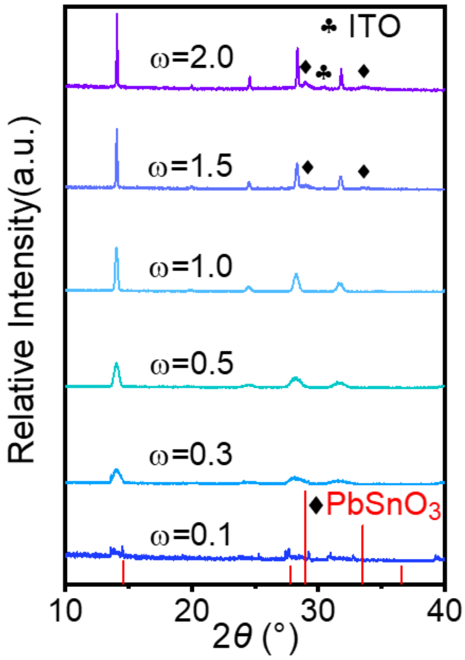

Supplementary Figure 6. GIXRD patterns of the top surface for K_2SnO_3-incorporated perovskite film (The square symbol represents $PbSnO_3$, and the club symbol represents ITO.).

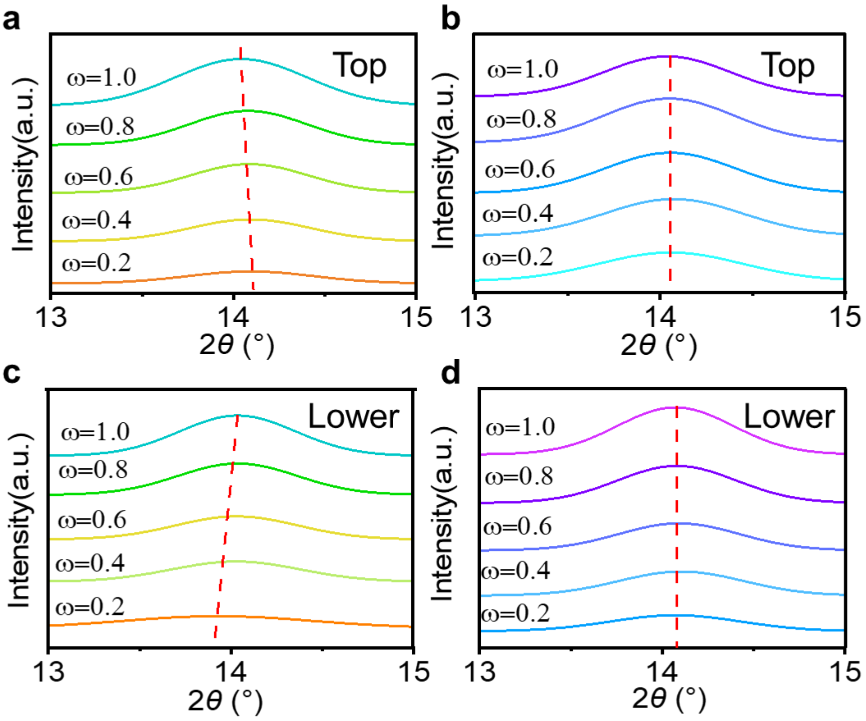

Supplementary Figure 7. GIXRD patterns of the top surface for (a) control and (b) K_2SnO_3-treated perovskite films. GIXRD patterns of the buried surface for (c) control and (d) K_2SnO_3-treated perovskite films.

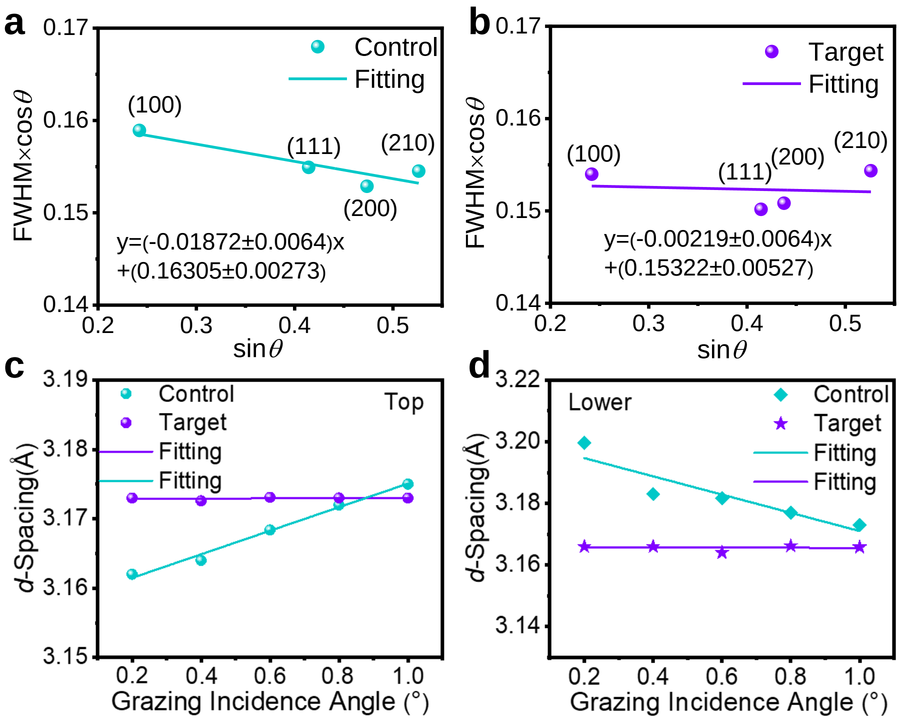

Supplementary Figure 8. Williamson-Hall diagrams of (a) control and (b) target films. D-spacing values of (c) the top surface and (d) the buried surface of the (100) plane as a function of the grazing-incidence angle.

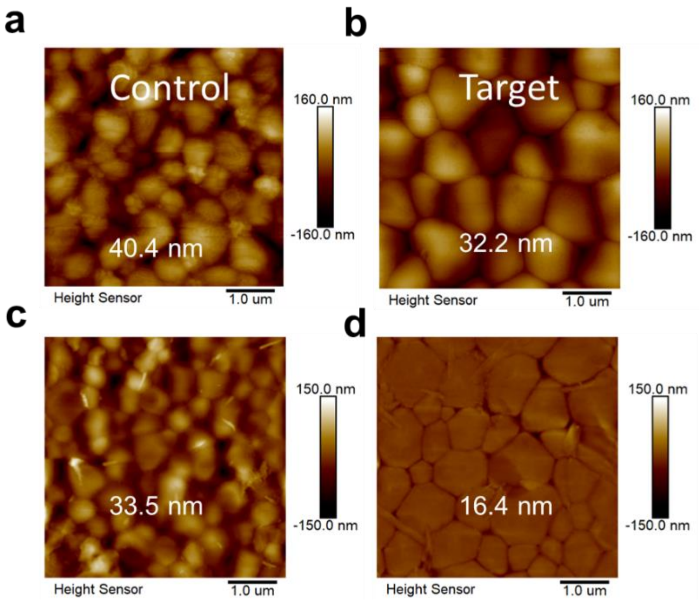

Supplementary Figure 9. AFM images of the top surface of (a) control and (b) target $FA_{0.7}MA_{0.3}Sn_{0.5}Pb_{0.5}I_3$ perovskite films. AFM images of the buried surface of (c) control and (d) target $FA_{0.7}MA_{0.3}Sn_{0.5}Pb_{0.5}I_3$ perovskite films.

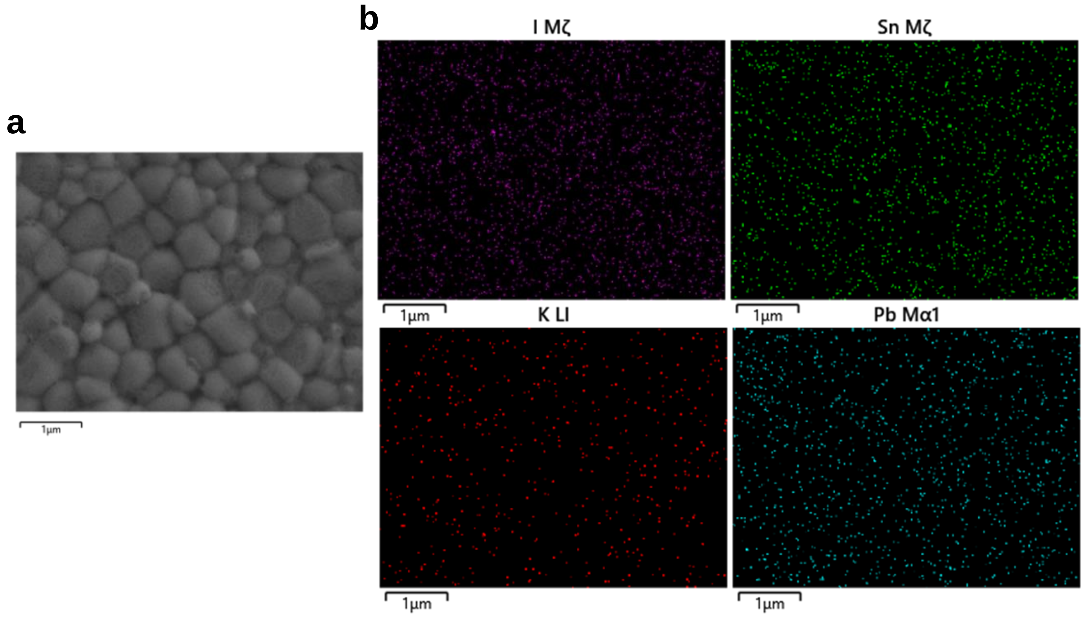

Supplementary Figure 10. (a) SEM image of a K_2SnO_3-modified perovskite film (b) with corresponding EDS elemental mapping, illustrating the distribution of key elements across the film.

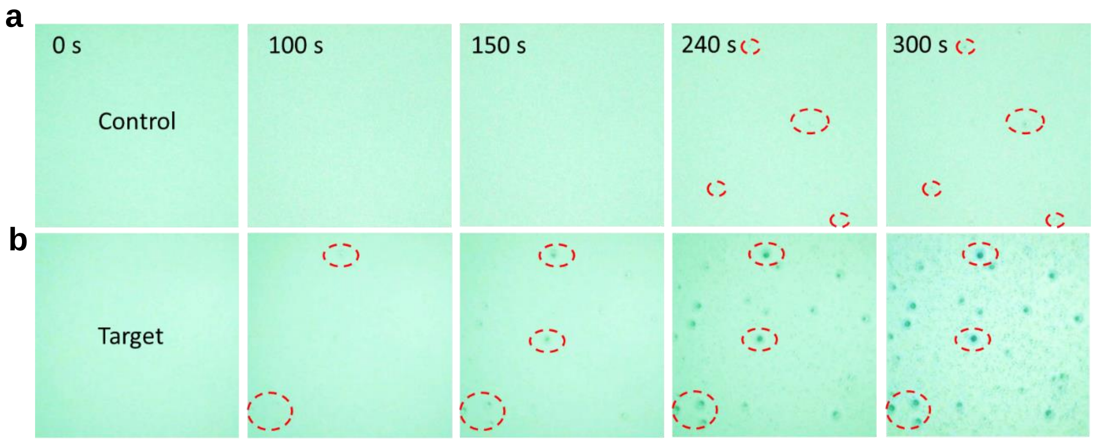

Supplementary Figure 11. Morphological evolution of perovskite precursor films (a) without and (b) with K_2SnO_3 during spin-coating in the absence of an anti-solvent. Optical microscope images were taken during the process.

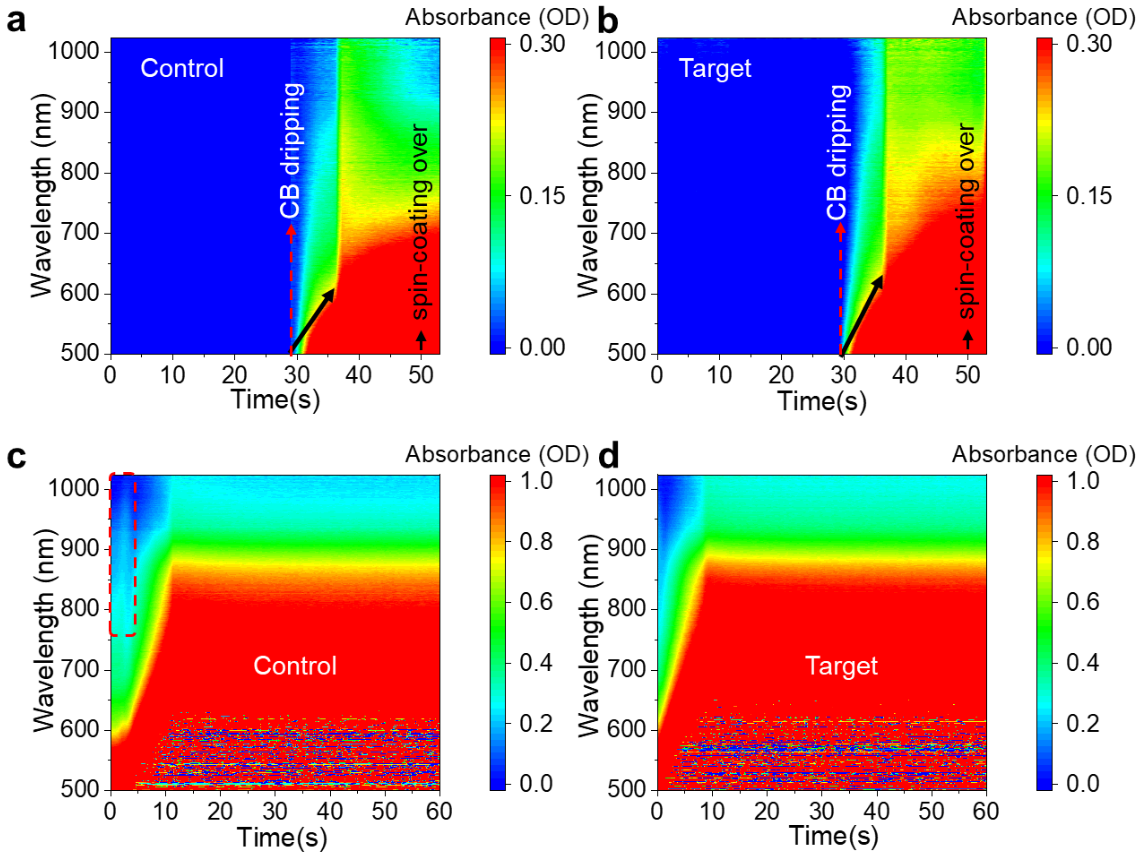

Supplementary Figure 12. In-situ light absorption evolution of Sn-Pb perovskite films, without and with K_2SnO_3 modification, during (a) (b) spin-coating and (c) (d) annealing stages.

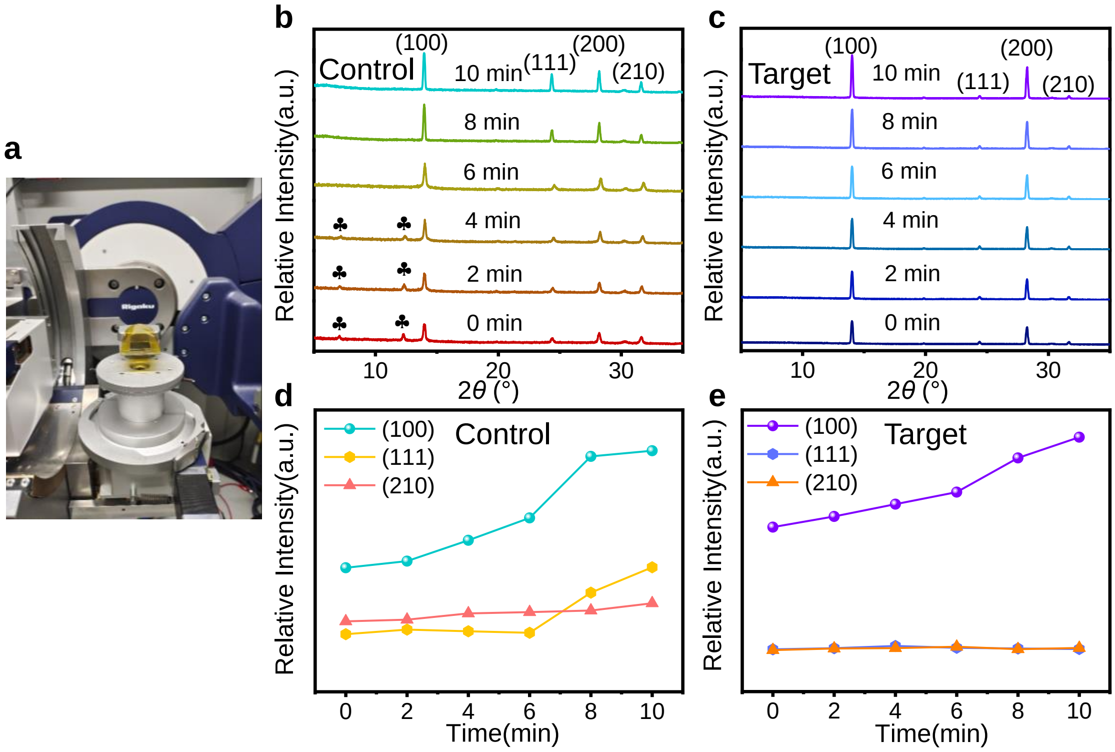

Supplementary Figure 13. (a) Photograph of the sample setup for time-tracked XRD testing. The samples were placed in a sealed box to prevent exposure to water and oxygen in the air. XRD patterns of Sn-Pb perovskite wet films prepared (b) without and (c) with K_2SnO_3 treatment, showing time evolution(The club symbol: perovskite intermediate phase.). (d) and (e) Diffraction peak intensity versus time for different crystal planes in (b) and (c), respectively.

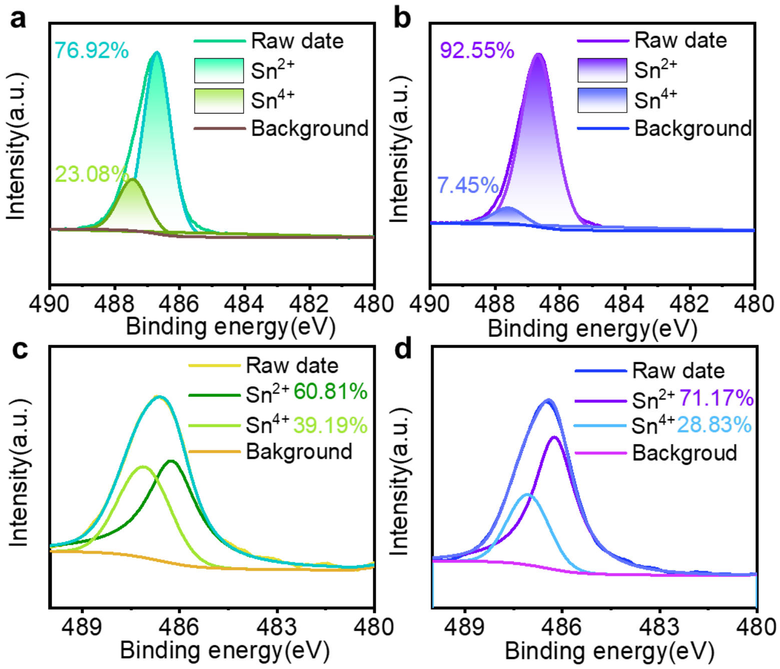

Supplementary Figure 14. XPS spectra of Sn 3*d* for the top surface of (a) control and (b) target perovskite films. XPS spectra of Sn 3*d* for the buried surface of (c) control and (d) target perovskite films.

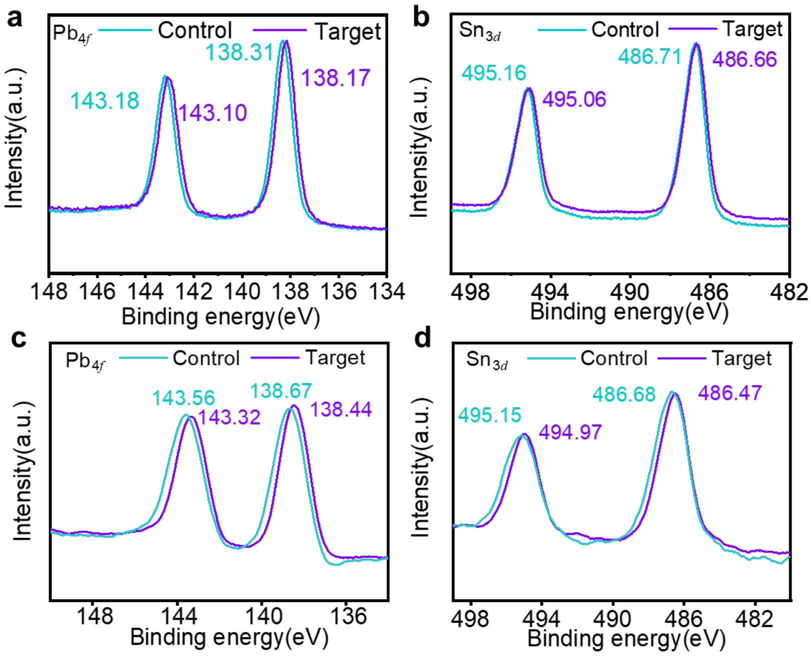

Supplementary Figure 15. XPS spectra of (a) Pb_{4f} and (b) Sn_{3d} of perovskite films on the top surface. XPS spectra of (c) Pb_{4f} and (d) Sn_{3d} of perovskite films on the bottom surface.

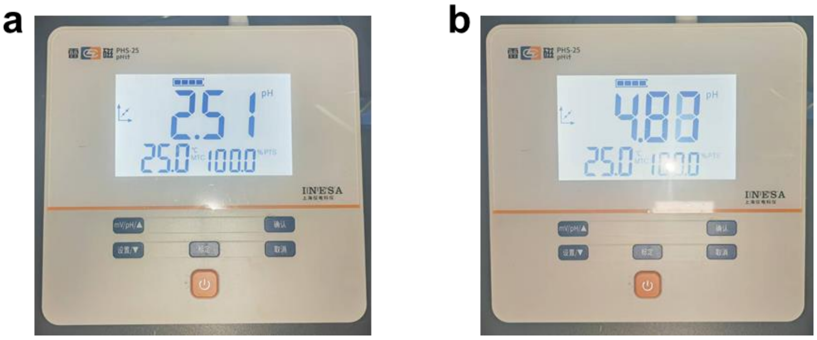

Supplementary Figure 16. pH values of (a) a PEDOT: PSS solution and (b) a PEDOT: PSS solution with K_2SnO_3 (3 mg mL^{-1}).

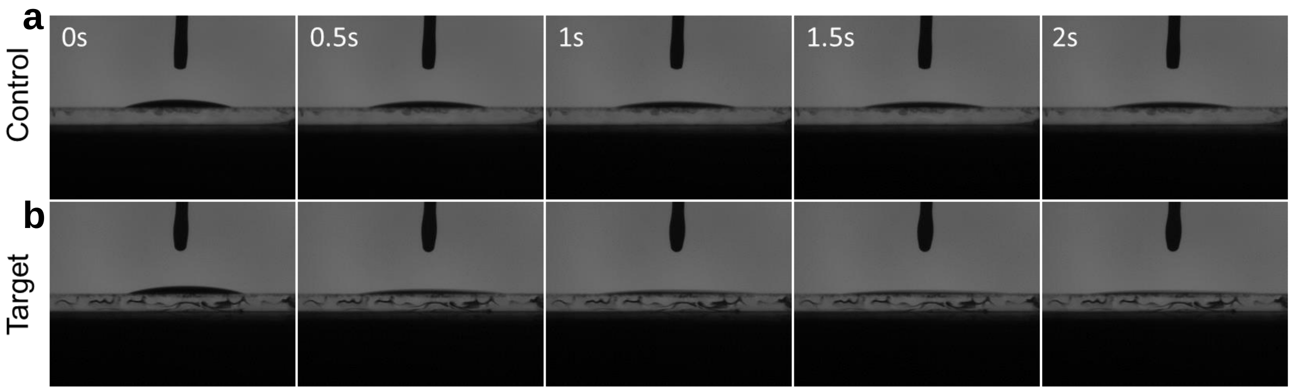

Supplementary Figure 17. (a)(b)Optical images of contact angle measurements of Sn-Pb perovskite precursor solutions on ITO/PEDOT: PSS substrates (Control: PEDOT: PSS, Target: PEDOT: PSS+3 mg mL^{-1} K_2SnO_3).

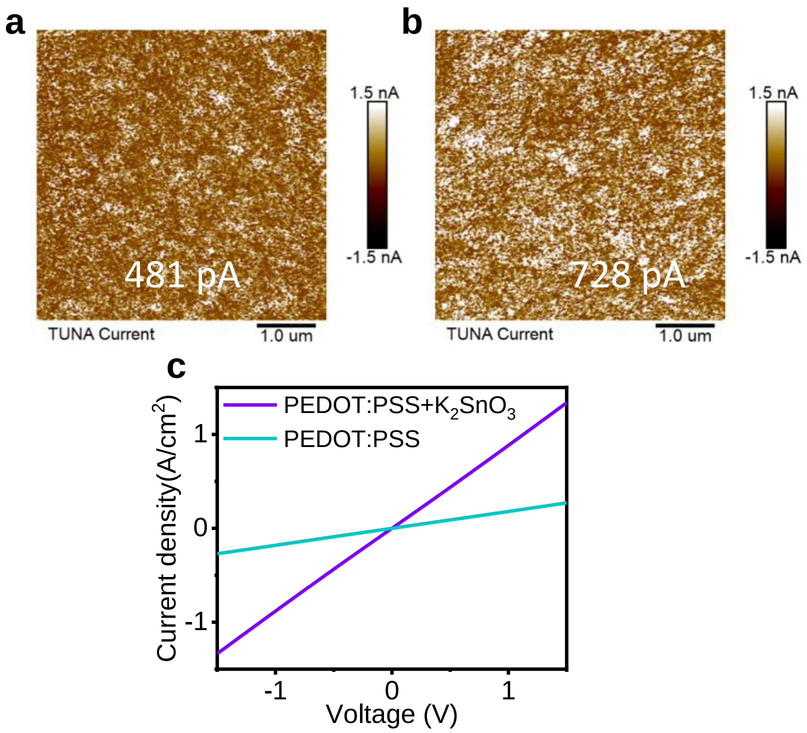

Supplementary Figure 18. Conductive AFM images of PEDOT: PSS (a) without and (b) with K_2SnO_3 modification. (c) Electrical conductivity measurements of PEDOT: PSS and PEDOT: PSS+3 mg mL^{-1} K_2SnO_3 HTLs with a structure of ITO/HTL/Cu.

As shown in Supplementary Figure 18a-b, the conductive AFM results indicated that the introduction of K_2SnO_3 effectively enhanced the conductivity of the PEDOT: PSS film, increasing from 481 pA in the control group to 728 pA. Additionally, based on the *J*-*V* test results, the conductivity of the PEDOT: PSS film increased from 5.4×10^{-4} to 2.65×10^{-3} S cm^{-1}, as calculated using Formula (1) (Supplementary Figure 18c).

$$I=\sigma_0(A/d)V \quad (1)$$

Where A, d, and σ_0 are the sample area (0.070225 cm^2), thickness (30 nm), and electrical conductivity, respectively.

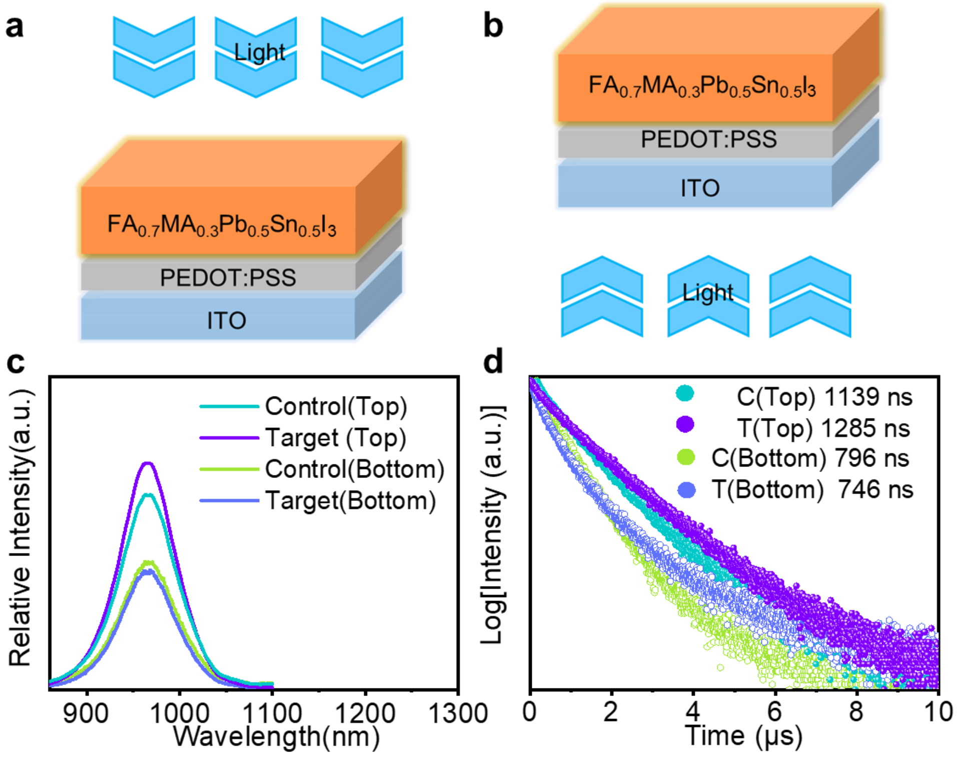

Supplementary Figure 19. Schematic diagram of PL measurements with excitation from the (a) top and (b) bottom of the films. (c) Steady-state and (d) time-resolved PL (TRPL) spectra of pristine Sn-Pb perovskite films on PEDOT: PSS/ ITO substrates (Control: PEDOT: PSS, Target: PEDOT: PSS+3 mg mL^{-1} K_2SnO_3). Target: K_2SnO_3 was incorporated into the PEDOT: PSS but not into the perovskite precursors. All films used for testing were top-capped with a polymethyl methacrylate thin film.

When excitation light was incident from the upper surface of the pristine perovskite film, the target group exhibited stronger PL intensity and a longer carrier lifetime. When excited from the bottom ITO glass side, the target film showed relatively weaker PL intensity and a shorter carrier lifetime due to more efficient hole extraction (Supplementary Figure 19d, Supplementary Table 2). This improvement is attributed to the incorporation of K_2SnO_3, which enhances the conductivity of PEDOT: PSS and facilitates carrier transport at the buried interface.

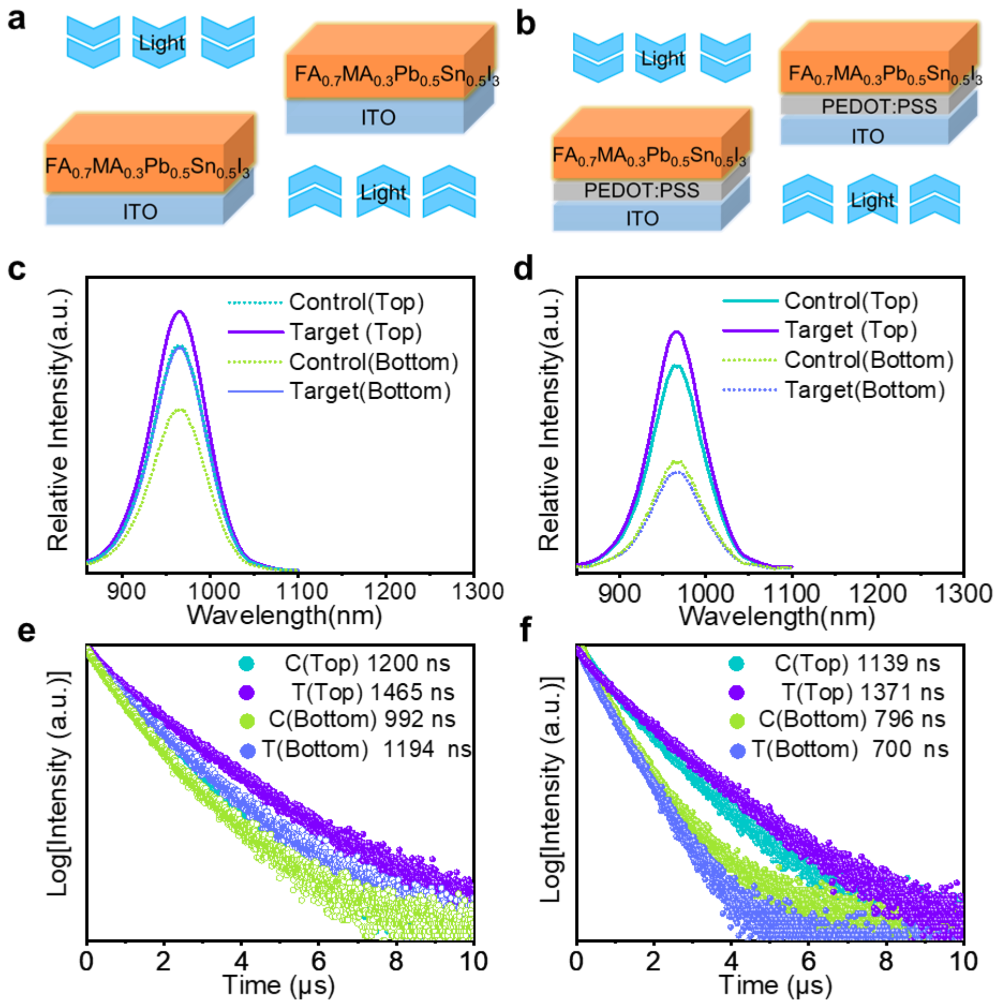

Supplementary Figure 20. (a-b) Schematic diagram of PL measurements with excitation from the top and bottom of the films. (c) Steady-state and (e) time-resolved PL spectra of control and K_2SnO_3-treated Sn-Pb perovskite films deposited on ITO substrates. (d) Steady-state and (f) TRPL spectra of control and target Sn-Pb perovskite films deposited on HTL-coated ITO substrates. Target: K_2SnO_3 was incorporated into both the perovskite precursors and PEDOT: PSS. All films used for testing were top-capped with a polymethyl methacrylate thin film.

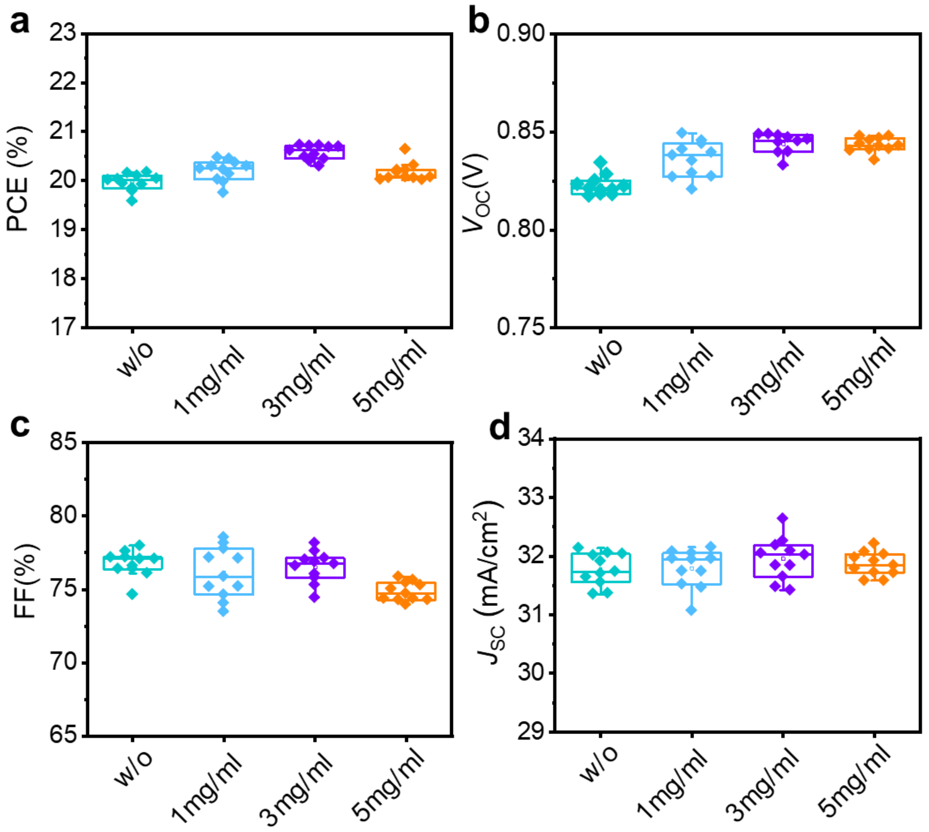

Supplementary Figure 21. Statistical diagram of device performance parameters with various amounts of K_2SnO_3 added to PEDOT: PSS solutions: (a) PCE, (b) V_{OC}, (c) FF, and (d) J_{SC}.

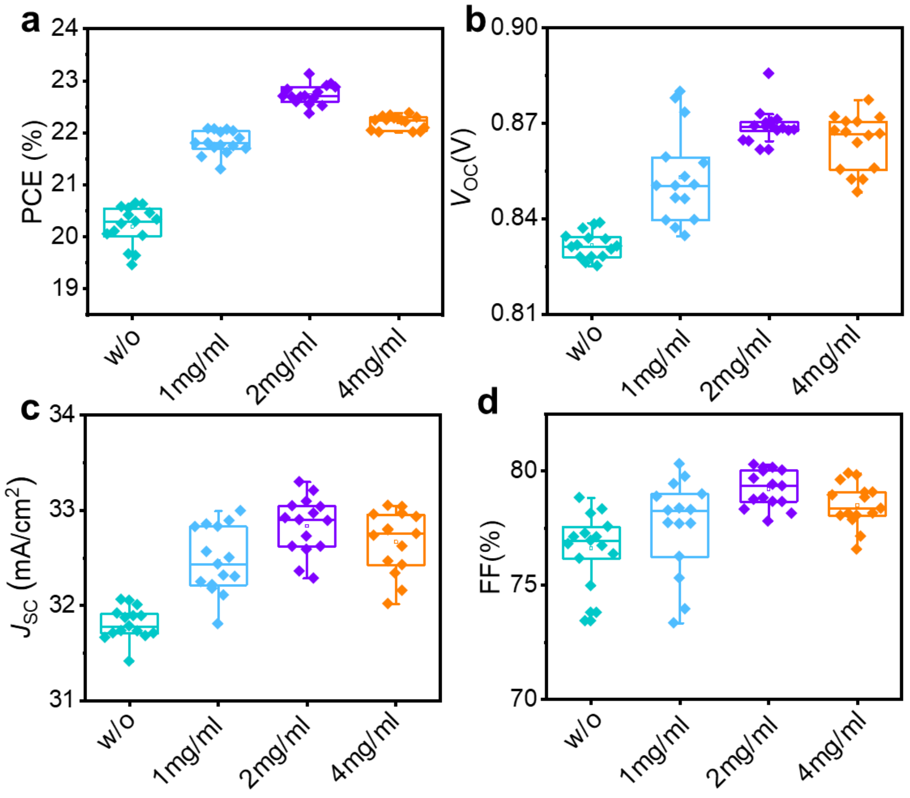

Supplementary Figure 22. Statistical diagram of device performance parameters with 3 mg mL^{-1} of K_2SnO_3 in PEDOT: PSS solutions and 0-4 mg mL^{-1} of K_2SnO_3 in perovskite precursor solutions: (a) PCE, (b) V_{OC}, (c) FF, and (d) J_{SC}.

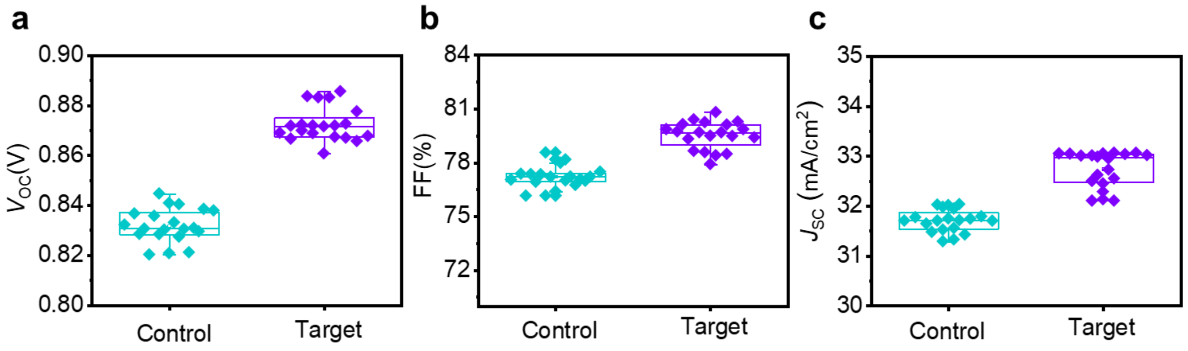

Supplementary Figure 23. Statistical data distribution of (a) V_{OC}; (b) FF; and (c) J_{SC} for control and target single-junction mixed Sn-Pb PSCs (20 devices for each type).

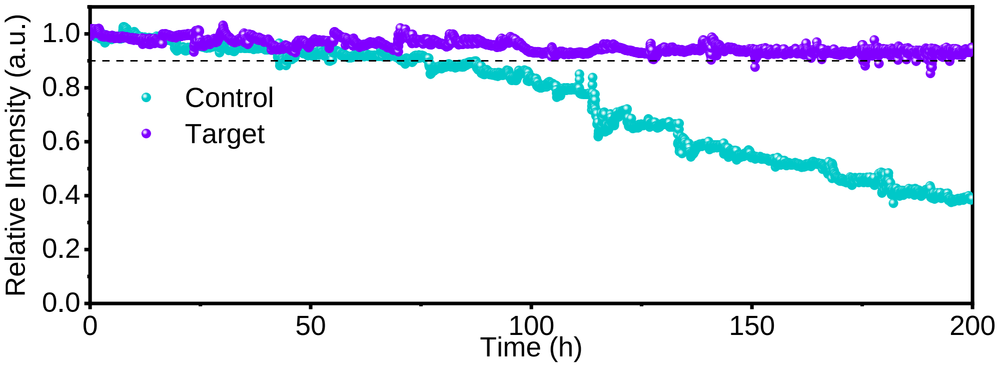

Supplementary Figure 24. Long-term stability tests of unencapsulated control and target Sn-Pb PSCs under MPP tracking with constant 1 sun illumination (using a light-emitting diode light source, equivalent to the global horizontal irradiance spectrum AM 1.5 G, 100 mW cm^{-2}) illumination in an N_2-filled glovebox at approximately 55°C.

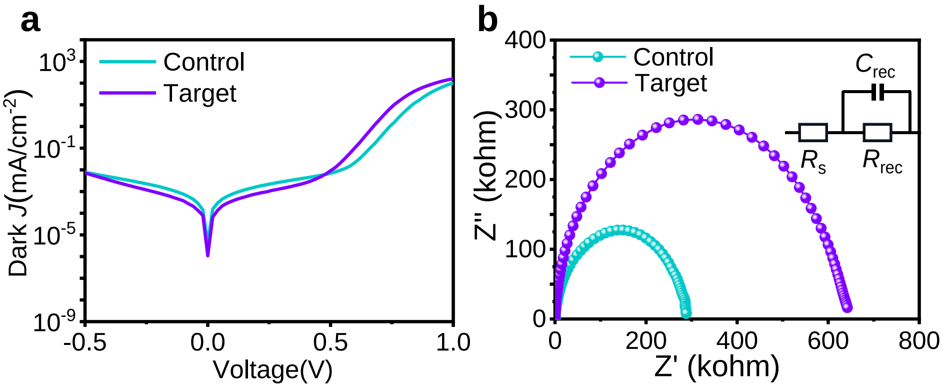

Supplementary Figure 25. (a) Dark *J-V* curves, (b) Nyquist plots of Sn-Pb perovskites without and with K_2SnO_3 modification.

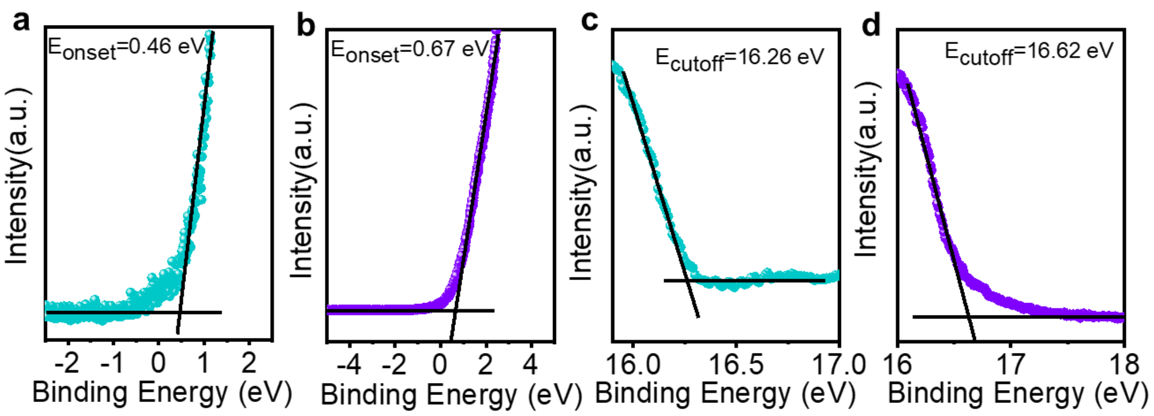

Supplementary Figure 26. (a) (b) Valence band regions and (c) (d) secondary electron cut-off of the UPS spectra for Sn-Pb perovskite films with or without K_2SnO_3 treatment.

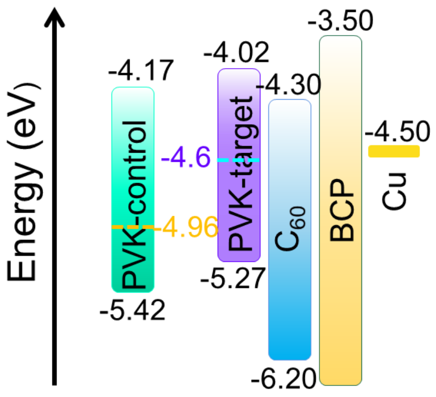

Supplementary Figure 27. Energy band alignment of Sn-Pb perovskites without and with K_2SnO_3 modification.

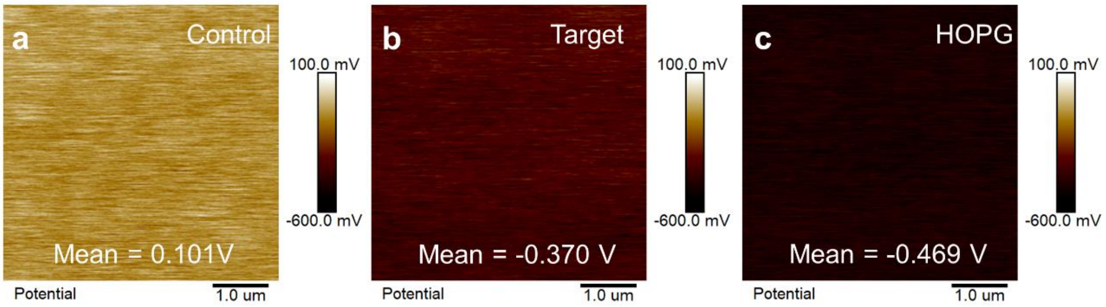

Supplementary Figure 28. KPFM images of perovskite films (a) without and (b) with K_2SnO_3 treatment. (c) KPFM image of a highly oriented pyrolytic graphite (HOPG) used as a standard sample.

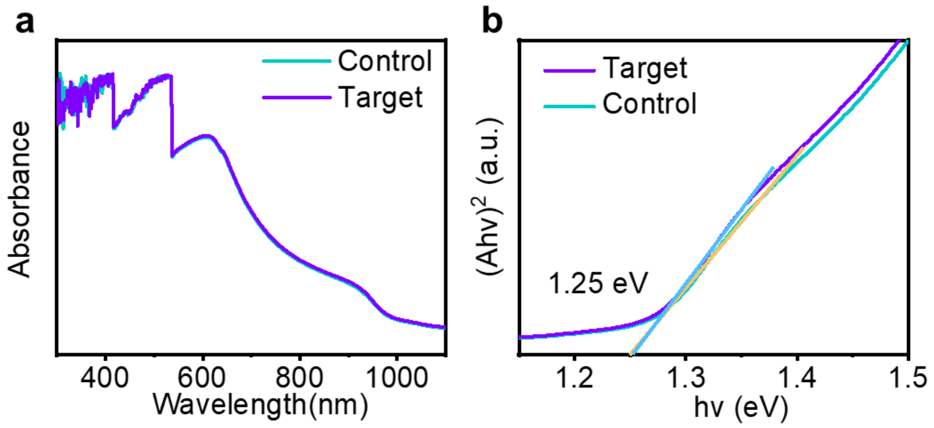

Supplementary Figure 29. (a) UV-Vis-NIR absorption spectra of control and target Sn-Pb perovskite films. (b) Tauc plots of Sn-Pb perovskite films with or without K_2SnO_3 treatment.

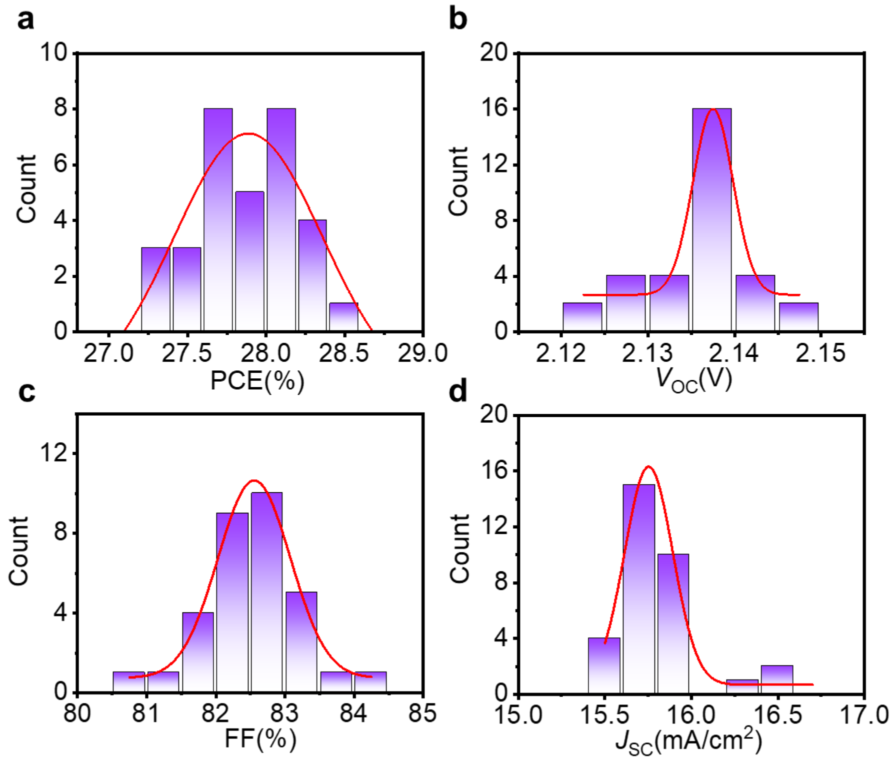

Supplementary Figure 30. Statistical data for all-perovskite tandem solar cells (TSCs): (a) PCE, (b) FF, (c) V_{OC}, and (d) J_{SC}. The average performance metrics are: PCE of 27.87± 0.31%, FF of 82.55 ± 0.68%, V_{OC} of 2.14 ± 0.01V, and J_{SC} of 15.81 ± 0.24 mA/cm^2 (32 devices for each type).

中国认可
国际互认
检测
TESTING
CNAS L8490

Test and Calibration Center of New Energy Device and Module,
Shanghai Institute of Microsystem and Information Technology,
Chinese Academy of Sciences (SIMIT)

Measurement Report

Report No. 24TR060102

**Client Name**	Wuhan University
**Client Address**	299 Bayi Road, Wuchang District, Wuhan, Hubei Province
**Sample**	Perovskite/perovskite tandem solar cell
**Manufacturer**	Wuhan University
**Measurement Date**	3rd June, 2024

**Performed by:**	Qiang Shi *Qiang Shi*	**Date:** 03/06/2024
**Reviewed by:**	Wenjie Zhao *Wenjie Zhao*	**Date:** 03/06/2024
**Approved by:**	Yucheng Liu *Yucheng Liu*	**Date:** 03/06/2024

Address: No.235 Chengbei Road, Jiading, Shanghai **Post Code:**201800

E-mail: solarcell@mail.sim.ac.cn **Tel:** +86-021-69976905

The measurement report without signature and seal are not valid.
This report shall not be reproduced, except in full, without the approval of SIMIT.

Sample Information

Sample Type	Perovskite/perovskite tandem solar cell
Serial No.	C3-1-1#
Lab Internal No.	24060101-2#
Measurement Item	I-V characteristic
Measurement Environment	24.4±2.0°C, 41.1±5.0%R.H

Measurement of I-V characteristic

**Reference cell**	PVM1121
**Reference cell Type**	mono-Si, WPVS, calibrated by NREL (Certificate No. ISO 2098)
**Calibration Value/Date of Calibration for Reference cell**	143.95mA/ Feb. 2024
**Measurement Conditions**	Standard Test Condition (STC):<br>Spectral Distribution: AM1.5,<br>Irradiance: 1000±50W/m^2, Temperature: 25±2°C
**Measurement Equipment/ Date of Calibration**	AAA Steady State Solar Simulator (YSS-T155-2M) / July.2023<br>IV test system (ADCMT 6246) / June. 2023<br>Measuring Microscope (MF-B2017C) / July.2023<br>SR Measurement system (CEP-25ML-CAS) / May.2024
**Measurement Method**	I-V Measurement:<br>Logarithmic sweep in both directions (Voc to Isc and Isc to Voc) during one flash based on IEC 60904-1:2020;<br>Spectral Mismatch factor was calculated and I-V correction was performed according to IEC 60891.
**Measurement Uncertainty**	Area: 1.0%(k=2); Isc: 2.1%(k=2); Voc: 1.0%(k=2);<br>Pmax: 2.8%(k=2); Eff: 2.9%(k=2)

SIMIT

Report No. 24TR060102

====Measurement Results ====

	Forward Scan (Isc to Voc)	Reverse Scan (Voc to Isc)
Area	6.58 mm^2	
Isc	1.051 mA	1.068 mA
Voc	2.156 V	2.160 V
Pmax	1.757 mW	1.816 mW
Ipm	0.926 mA	0.960 mA
Vpm	1.896 V	1.892 V
FF	77.52 %	78.68 %
Eff	26.70 %	27.60 %

- Spectral Mismatch Factor SMM_{top}=1.0055, SMM_{bot}=0.9887.
- Designated Illumination area defined by a thin mask was measured by the measuring microscope.
- Test results listed in this measurement report refer exclusively to the mentioned measured sample.
- The results apply only at the time of the test, and do not imply future performance.

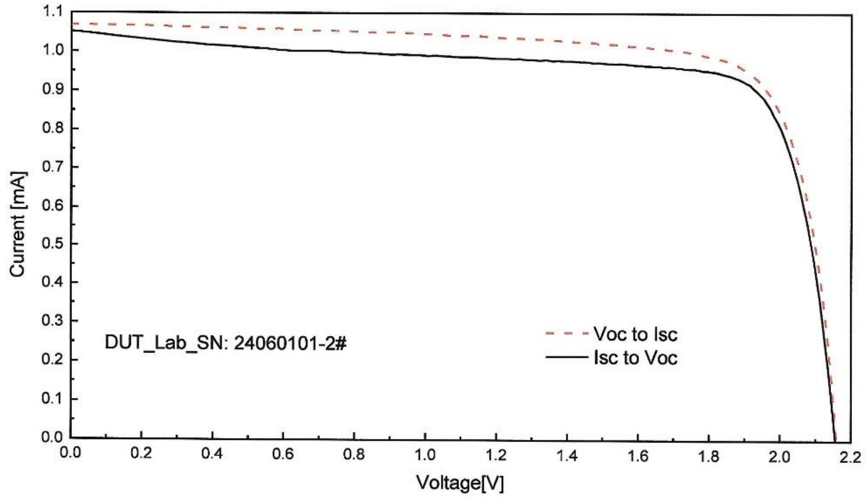

Fig.1 I-V curves of the measured sample

-------End of Report-------

3 / 3

Supplementary Figure 31. Certification report for a representative all-perovskite tandem cell, incorporating a K_2SnO_3-modified Sn-Pb subcell, issued by the Shanghai Institute of Microsystem and Information Technology (SIMIT).

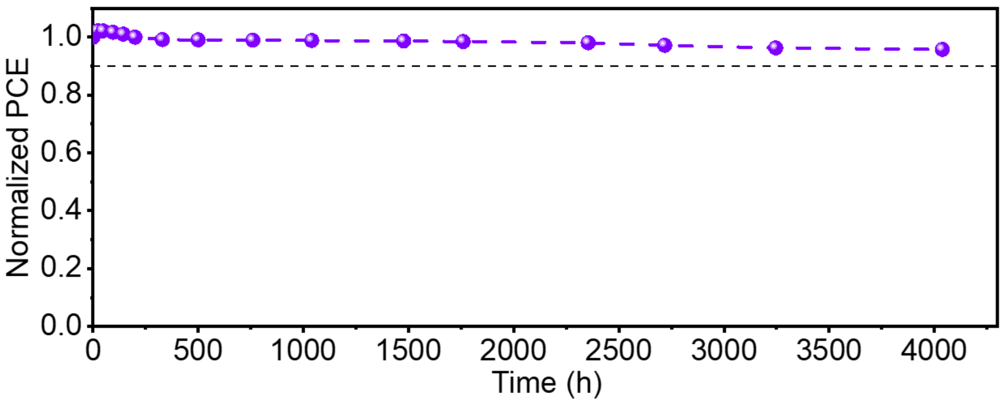

Supplementary Figure 32. Long-term storage stability of an unencapsulated tandem device stored in an N_2-filled glovebox at room temperature.

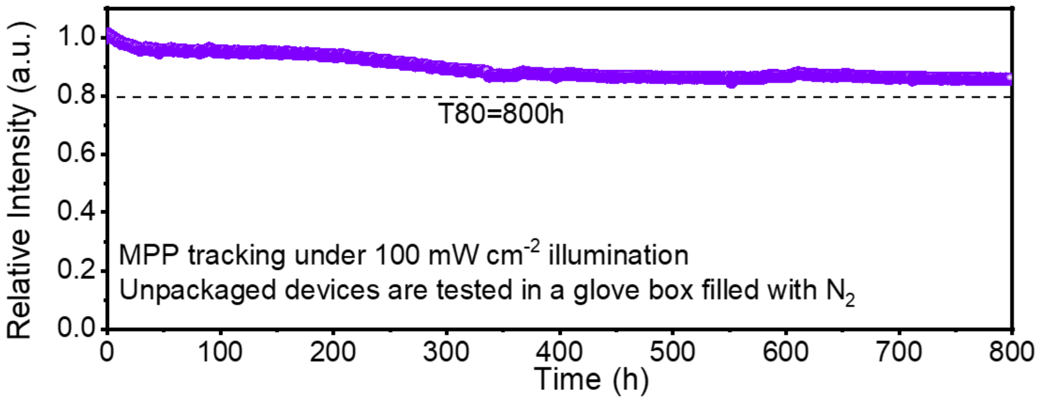

Supplementary Figure 33. Long-term MPP tracking of an unencapsulated all-perovskite tandem cell under constant 1 sun illumination in an N_2-filled glovebox at 55°C.

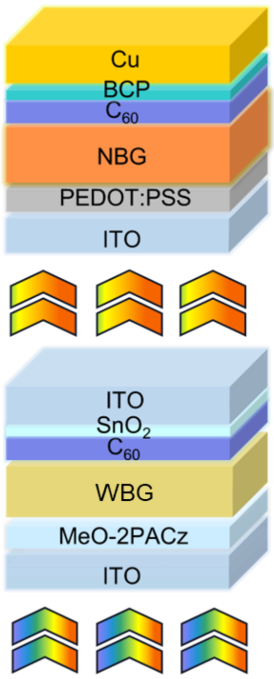

Supplementary Figure 34. Device architecture diagrams of perovskite/perovskite 4T TSCs.

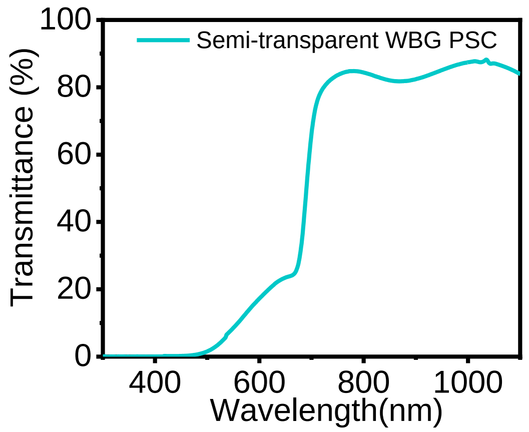

Supplementary Figure 35. Transmittance spectrum of a semi-transparent WBG PSC.

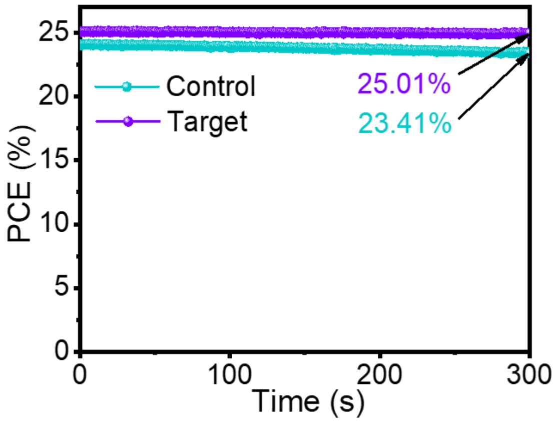

Supplementary Figure 36. SPO efficiencies of control and K_2SnO_3-modified single-junction normal-bandgap (1.54 eV) PSCs.

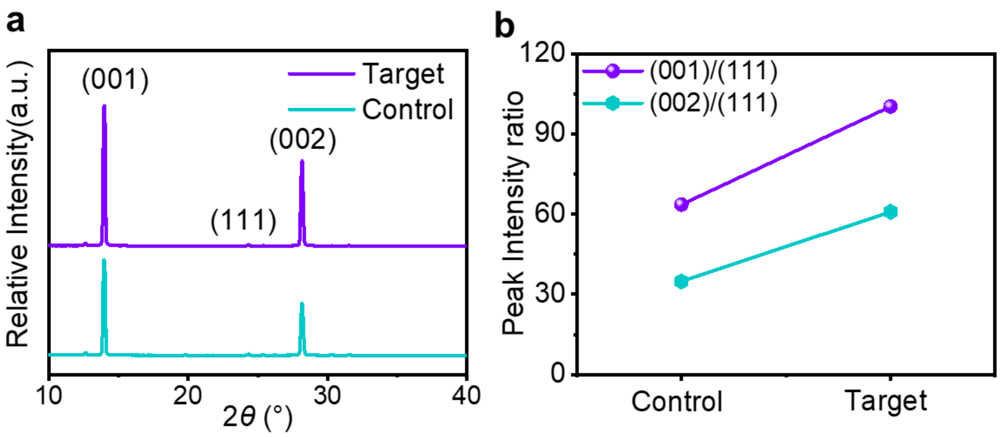

Supplementary Figure 37. (a) XRD patterns of control and K_2SnO_3-treated $FA_xMA_{1-x}PbI_3$ perovskite films. (b) Ratios of peak intensities from different crystal planes.

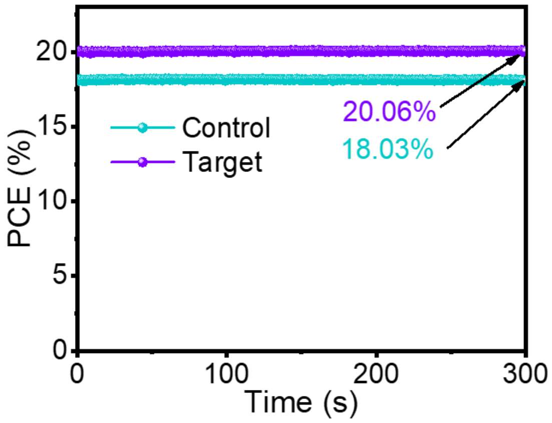

Supplementary Figure 38. SPO efficiencies of control and K_2SnO_3-modified single-junction WBG (1.77 eV) PSCs.

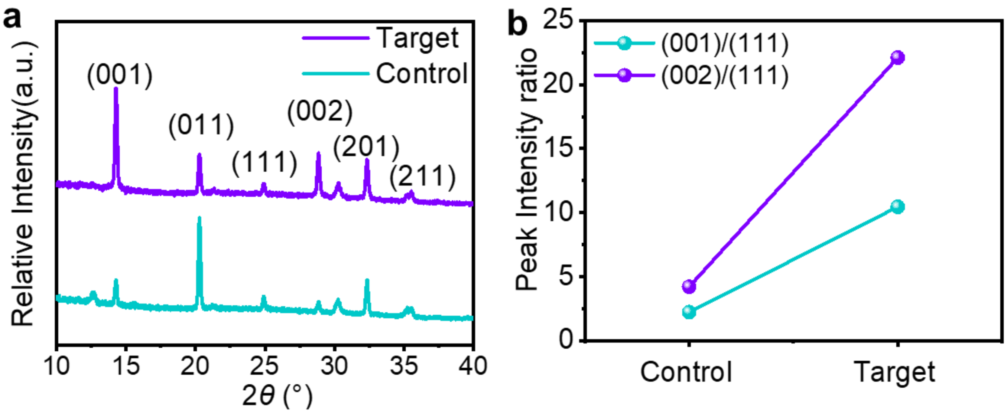

Supplementary Figure 39. (a) XRD patterns of control and K_2SnO_3-treated 1.77 eV WBG perovskite films. (b) Ratios of peak intensities from different crystal planes.

Supplementary Table 1. Calculation of lattice matching rate.

Sample	Main diffraction peak 2θ (°)	d-spacing (Å)	Lattice matching rate
$FA_{0.7}MA_{0.3}Pb_{0.5}Sn_{0.5}I_3$ (100)	14.02	3.18	97.80%
$PbSnO_3$ (111)	14.35	3.11	
$FA_{0.7}MA_{0.3}Pb_{0.5}Sn_{0.5}I_3$ (200)	28.23	1.63	98.16%
$PbSnO_3$ (222)	28.78	1.60	

Supplementary Table 2. TRPL fitting data of Sn-Pb perovskite films deposited on PEDOT: PSS/ ITO substrates (Control: PEDOT: PSS, Target: PEDOT: PSS+3 mg mL^{-1} K_2SnO_3).

Sample	A_1	τ_1 (ns)	A_2	τ_2 (ns)	τ_{avg} (ns)
Control (Top)	2620.3	949.7	544.0	2051.7	1139
Target (Top)	636.6	393.0	2326.1	1533.5	1285
Control(Bottom)	3280.4	736.0	44.7	4908.3	796
Target(Bottom)	1805.0	390.4	1102.4	1332.4	746

The relationship among τ_1, τ_2, and τ_{avg} is represented by the equation (2):

$$\tau_{avg} = \frac{A_1\tau_1+A_2\tau_2}{A_1+A_2} \tag{2}$$

where A_1 and A_2 are relative amplitudes, and τ_1 and τ_2 represent the lifetimes for fast and slow recombination, respectively. τ_{avg} represent the average lifetime.

Supplementary Table 3. TRPL fitting data of control and target Sn-Pb perovskite films deposited on ITO substrates.

Sample	A_1	τ_1 (ns)	A_2	τ_2 (ns)	τ_{avg} (ns)
Control (Top)	1351.4	832.1	1666.0	1499.6	1200
Target (Top)	756.0	510.1	2287.9	1779.7	1465
Control(Bottom)	1847.17	692.2	1074.64	1508.9	992
Target (Bottom)	677.7	381.9	2241.8	1442.1	1194

Supplementary Table 4. TRPL fitting data of control and target Sn-Pb perovskite films deposited on PEDOT: PSS-coated ITO substrates. For the target samples, K_2SnO_3 was incorporated into both the perovskite precursors and PEDOT: PSS.

Sample	A_1	τ_1 (ns)	A_2	τ_2 (ns)	τ_{avg} (ns)
Control (Top)	2620.3	949.7	544.0	2051.7	1139
Target (Top)	568.1	364.0	2306.5	1622.8	1371
Control(Bottom)	3280.4	736.0	44.7	4908.3	796
Target(Bottom)	561.6	344.4	2525.9	783.9	700

Supplementary Table 5. Photovoltaic parameters (open-circuit voltage (V_{OC}) ; short-circuit current density (J_{SC}); fill factor (FF); and power conversion efficiencie (PCE)) of the best-performing single-junction target $FA_{0.7}MA_{0.3}Pb_{0.5}Sn_{0.5}I_3$ PSC.

2.1 M $FA_{0.7}MA_{0.3}Pb_{0.5}Sn_{0.5}I_3$	V_{OC} (V)	J_{SC} (mA cm^{-2})	FF (%)	PCE (%)
Target (Forward)	0.88	33.07	78.56	22.97
Target (Reverse)	0.88	32.90	80.15	23.32
Taget SPO				23.12

Supplementary Table 6. Photovoltaic parameters of the best-performing 2T all-perovskite TSC.

2T all-perovskite TSC	V_{OC} (V)	J_{SC} (mA cm^{-2})	FF (%)	PCE (%)
Forward	2.14	15.82	82.34	27.85
Reverse	2.14	15.89	83.07	28.20
SPO				28.12

Supplementary Table 7. Photovoltaic parameters of a WBG subcell and a light-filtered NBG subcell within a 4T all-perovskite TSC measured under reverse voltage scans and their SPO efficiencies.

4T all-perovskite TSC	V_{OC} (V)	J_{SC} (mA cm^{-2})	FF (%)	PCE (%)
WBG (*J*-*V*)	1.30	16.85	83.87	18.39
WBG SPO				18.36
NBG (*J*-*V*)	0.85	15.09	81.64	10.52
NGB SPO				10.45
4T (*J*-*V*)				28.91
4T SPO				28.81

Supplementary Table 8. Detailed *J*-*V* parameters of the 1.54 eV normal-bandgap devices with or without 1 mg mL^{-1} K_2SnO_3 in PbI_2 precursor solutions.

1.54 eV $FA_xMA_{1-x}PbI_3$	V_{OC} (V)	J_{SC} (mA cm^{-2})	FF (%)	PCE (%)
Control Forward	1.187	25.56	78.30	23.77
Control Reverse	1.188	25.18	80.39	24.05
Target Forward	1.189	25.50	82.06	24.89
Target Reverse	1.193	25.57	82.26	25.09

Supplementary Table 9. Detailed *J-V* parameters of the 1.77 eV WBG devices with or without K_2SnO_3 in precursor solutions.

1.77 eV $FA_{0.8}Cs_{0.2}Pb(I_{0.6}Br_{0.4})_3$	V_{OC} (V)	J_{SC} (mA cm^{-2})	FF (%)	PCE (%)
Control Forward	1.30	18.18	76.58	18.06
Control Reverse	1.29	18.20	77.46	18.25
Target Forward	1.31	18.58	79.62	20.00
Target Reverse	1.31	18.65	79.94	20.13

Supplementary References

[1] Jung, M., Ji, S.-G., Kim, G. & Seok, S. I. Perovskite precursor solution chemistry: from fundamentals to photovoltaic applications. *Chem. Soc. Rev.* **48**, 2011-2038 (2019).

[2] Zhang, H. *et al.* Controllable Heterogenous Seeding-Induced Crystallization for High-Efficiency $FAPbI_3$-Based Perovskite Solar Cells Over 24%. *Adv. Mater.* **34**, 2204366 (2022).
