# Supplementary material for: Universal in situ oxide-based ABX3-structured seeds for templating halide perovskite growth in All-perovskite tandems
Source: Nat Commun. 2025 Feb 22;16:1894. doi: 10.1038/s41467-025-57195-w (PMC11846837; doi:10.1038/s41467-025-57195-w)
Supplement: Supplementary file 2 — reporting summary [file 41467_2025_57195_MOESM2_ESM.pdf]

Corresponding author(s): Weijun Ke

Solar Cells Reporting Summary

Nature Portfolio wishes to improve the reproducibility of the work that we publish. This form is intended for publication with all accepted papers reporting the characterization of photovoltaic devices and provides structure for consistency and transparency in reporting. Some list items might not apply to an individual manuscript, but all fields must be completed for clarity.

For further information on Nature Research policies, including our data availability policy, see Authors & Referees.

Experimental design

Please check the following details are reported in the manuscript, and provide a brief description or explanation where applicable.

1. Dimensions

Area of the tested solar cells

- [x] Yes — Methods Section, Device performance measurements. "The device area was 0.0948 cm2"
- [ ] No — *Explain why this information is not reported/not relevant.*

Method used to determine the device area

- [x] Yes — Methods Section, Device performance measurements. "The device area was 0.0948 cm2 and masked with a metal aperture to define an active area of 0.070225 cm2."
- [ ] No — *Explain why this information is not reported/not relevant.*

2. Current-voltage characterization

Current density-voltage (J-V) plots in both forward and backward direction

- [x] Yes — Figure 4a, Figure 5b, 5e,5h,5i and Supplementary Table 5,6,7,8, and 9.
- [ ] No

Voltage scan conditions

- [x] Yes — Methods Section, Device performance measurements. "The J–V curves of both single-junction and tandem solar cells were taken with reverse and forward voltage scans with a scan speed of 0.02 V/s, a voltage step of 20 mV, and a delay time of 25 ms."
- [ ] No — *Explain why this information is not reported/not relevant.*

Test environment

- [x] Yes — At room temperature and in an N2-filled glove box.
- [ ] No — *Explain why this information is not reported/not relevant.*

Protocol for preconditioning of the device before its characterization

- [ ] Yes — *Provide a description of the protocol.*
- [x] No — No preconditioning is required for our cell characterization

Stability of the J-V characteristic

- [x] Yes — Figure 4c, Figure 5c,5f and Supplementary Figure 36, Figure 38, stable power output efficiencies near the maximum power point voltages were provided.
- [ ] No — *Explain why this information is not reported/not relevant.*

3. Hysteresis or any other unusual behaviour

Description of the unusual behaviour observed during the characterization

- [x] Yes — Negligible hysteresis was found.
- [ ] No — *Explain why this information is not reported/not relevant.*

Related experimental data

- [x] Yes — J-V curves under reverse and forward scans were provided.
- [ ] No — *Explain why this information is not reported/not relevant.*

4. Efficiency

External quantum efficiency (EQE) or incident photons to current efficiency (IPCE)

- [x] Yes — EQE curves were provided in Figure 4b, and Figure 5d,5g.
- [ ] No — *Explain why this information is not reported/not relevant.*

A comparison between the integrated response under the standard reference spectrum and the response measure under the simulator

☒ Yes ☐ No

The integrated Jsc values obtained from EQE were agree well with the Jsc determined from the J–V measurements

Explain why this information is not reported/not relevant.

For tandem solar cells, the bias illumination and bias voltage used for each subcell

☒ Yes ☐ No

Methods Section, Device performance measurements.

Explain why this information is not reported/not relevant.

5. Calibration

Light source and reference cell or sensor used for the characterization

☒ Yes ☐ No

Methods Section, Device performance measurements.

Explain why this information is not reported/not relevant.

Confirmation that the reference cell was calibrated and certified

☒ Yes ☐ No

Methods Section, Device performance measurements. "The reference cell was certified by the certified WPVS standard solar reference cell (SRC-2020, Enlitech; traceable to NREL) at 100mW/cm2"

Explain why this information is not reported/not relevant.

Calculation of spectral mismatch between the reference cell and the devices under test

☐ Yes ☒ No

Provide a value of the spectral mismatch and/or a description of how it has been taken into account in the measurements.

The light spectrum used for measurements matches well with the reference silicon cell, and we did not calculate the spectral mismatch between the reference cell and the tested devices.

6. Mask/aperture

Size of the mask/aperture used during testing

☒ Yes ☐ No

0.070225 cm2

Explain why this information is not reported/not relevant.

Variation of the measured short-circuit current density with the mask/aperture area

☒ Yes ☐ No

Negligible

Explain why this information is not reported/not relevant.

7. Performance certification

Identity of the independent certification laboratory that confirmed the photovoltaic performance

☒ Yes ☐ No

A representative single-junction solar cell and a tandem cell were certified by Shanghai Institute of Measurement and Testing Technology (SIMT)

Explain why this information is not reported/not relevant.

A copy of any certificate(s)

☒ Yes ☐ No

Supplementary Fig. 31

Explain why this information is not reported/not relevant.

8. Statistics

Number of solar cells tested

☒ Yes ☐ No

Thirty-two for tandem devices

Explain why this information is not reported/not relevant.

Statistical analysis of the device performance

☒ Yes ☐ No

Figure 4d and Supplementary Figure 21, 22, 23, 30.

Explain why this information is not reported/not relevant.

9. Long-term stability analysis

Type of analysis, bias conditions and environmental conditions

☒ Yes ☐ No

Figure 4e, Supplementary Figure 24,32,33, and their legends, Methods Section, Device performance measurements.

Explain why this information is not reported/not relevant.
